# Impact of Cooking on Bioactive Compounds and Antioxidant Activity of Pigmented Rice Cultivars

**DOI:** 10.3390/foods9080967

**Published:** 2020-07-22

**Authors:** Daniela Fracassetti, Carola Pozzoli, Sara Vitalini, Antonio Tirelli, Marcello Iriti

**Affiliations:** 1 Department of Food, Environmental and Nutritional Sciences, Università degli Studi di Milano, Via G. Celoria 2, 20133 Milan, Italy; daniela.fracassetti@unimi.it (D.F.); carola.pozzoli@unimi.it (C.P.); antonio.tirelli@unimi.it (A.T.); 2 Department of Agricultural and Environmental Sciences, Production, Landscape, Agroenergy, Università degli Studi di Milano, Via G. Celoria 2, 20133 Milan, Italy; sara.vitalini@unimi.it

**Keywords:** Venere rice, Artemide rice, phenols, flavonoids, anthocyanins, antiradical

## Abstract

Pigmented rice cultivars, namely Venere and Artemide, are a source of bioactive molecules, in particular phenolics, including anthocyanins, exerting a positive effect on cardiovascular systems thanks also to their antioxidant capacity. This study aimed to determine the total phenol index (TPI), total flavonoids (TF), total anthocyanins (TA) and in vitro antioxidant capacity in 12 batches of Venere cultivar and two batches of Artemide cultivar. The rice was cooked using different methods (boiling, microwave, pressure cooker, water bath, rice cooker) with the purpose to individuate the procedure limiting the loss of bioactive compounds. TPI, TF and TA were spectrophotometrically determined in both raw and cooked rice samples. Rice samples of Artemide cultivars were richer in TPI (17.7–18.8 vs. 8.2–11.9 g gallic acid/kg in Venere rice), TF (13.1 vs. 5.0–7.1 g catechin/kg rice for Venere rice) and TA (3.2–3.4 vs. 1.8–2.9 g Cy-3glc/kg for Venere rice) in comparison to those of Venere cultivar; as well, they showed higher antioxidant capacity (46.6–47.8 vs. 14.4–31.9 mM Trolox/kg for Venere rice). Among the investigated cooking methods, the rice cooker and the water bath led to lower and comparable losses of phenolics. Interestingly, the cooking water remaining after cooking with the rice cooker was rich in phenolics. The consumption of a portion of rice (100 g) cooked with the rice cooker with its own cooking water can supply 240 mg catechin and 711 mg cyanidin 3-*O*-glucoside for Venere rice and 545 mg catechin and 614 mg cyanidin 3-*O*-glucoside for Artemide rice, with a potential positive effect on health.

## 1. Introduction

Rice (*Oryza sativa* L.) is among the most highly consumed food worldwide. The white genotype is the most common one even if pigmented varieties have been gaining increasing interest. They are characterized by a peculiar black, red or brown grain, due to the presence of natural pigments in the pericarp, seminal integument and nucellar layer of the caryopsis. Pigmented rice is traditionally cultivated and consumed in Asian countries, where rice represents one of the main sources of energy, due to its high carbohydrate content [1]. More recently, the interest in pigmented rice and its consumption have also grown in Western countries. This can be attributed to the present of bioactive compounds, including anthocyanins, flavonoids and phenolic acids [2,3,4,5,6]. Thanks to these molecules, several biological activities associated with rice consumption were shown, such as antiatherosclerosis [7,8,9], antioxidant, anti-inflammatory and inhibitory properties against α-glucosidase and α-amylase [10].

Rice can be cooked in many different ways, at home or on an industrial scale, in terms of tools used, temperatures and times of preparation. The two main cooking methods include boiling in an excess of water and cooking by water absorption (pilaf method). The first method is widely used in Western countries and in industrial preparations, while the second is commonly employed in Asian countries and often combined with an electric rice cooker. Precooking (washing and/or soaking) and postcooking processes (storage, freezing, drying, freeze drying, sterilization) can also be performed. Cooking has a direct impact on the behavior of the rice grains during thermal treatment and on the final quality of the product. The lack of homogeneity of methods and timing is particularly critical in the preparation of pigmented rice, which is naturally rich in bioactive compounds. The loss of phenolic compounds after cooking can be attributable to multiple factors, including the water–rice ratio, cooking times and temperatures. The main methods reported for cooking pigmented rice are boiling, frying or cooking in a water bath, with rice cooker or pressure cooker [11,12,13,14,15]. Zaupa et al. [6] suggested that water bath cooking, defined as “risotto” cooking, is the cooking method that allows preserving both its phenolic compounds and total antioxidant capacity. However, in the aforementioned study, the cooking of the pigmented rice was performed under laboratory scale (five grains), not reflecting the real domestic cooking of an average portion of consumption (70–100 g). Besides the cooking method, Abdel-Aal and Hucl [16] and Hiemori et al. [12] showed the cooking temperature affects the stability of anthocyanins in pigmented cereals including rice. Otherwise, no difference depending on the cooking temperature was found by Zaupa et al. [6], perhaps because the loss of anthocyanins in boiled rice could be ascribed to the used medium (water) [17].

To the best of our knowledge, little is known about the content of bioactive compounds and the antioxidant capacity in pigmented rice cultivars, including Venere and Artemide, which are cultivated and consumed in Italy. Moreover, the effect of different cooking procedures miming the preparation of a rice portion requires further clarification.

This study aimed to evaluate the content of anthocyanins, flavonoids and phenolics as well as the antioxidant capacity of Venere and Artemide rice cultivars by comparing them with those of Carnaroli brown rice. Moreover, different cooking methods were carried out in different conditions, in order to identify the optimal cooking conditions leading to the lowest possible loss of bioactive compounds.

## 2. Materials and Methods

### 2.1. Chemicals and Reagents

Standards of gallic acid, sodium acetate, 2,2′-azino-bis(3-ethylbenzothiazoline-6-sulphonic acid) (ABTS), 2,2-diphenyl-1-picrylhydrazyl (DPPH), Folin–Ciocalteau reagent, 6-hydroxy-2,5,7,8-tetramethylchroman-2-carboxylic acid (Trolox) and hydrochloric acid were purchased from Sigma-Aldrich (Darmstadt, Germany). Cyanidin 3-*O*-glucoside (Cy-3glc) was purchased from Extrasynthese (Genay, France). Ethanol and methanol were purchased from Novachimica (Milano, Italy).

### 2.2. Rice Cultivars

Three cultivars of whole pigmented rice were analyzed: a brown (Carnaroli) and two pigmented (Artemide and Venere) cultivars. The rice was cultivated in different fields in the same area (Vercelli, Italy) and they were collected in a farm after the harvest that was performed in 2018. Twelve different batches were provided for the Venere cultivar (labeled 1–12), two for the Artemide cultivar (labeled 13–14) and one for the Carnaroli cultivar (labeled 15). All fifteen samples were stored at 4 °C in the dark until analysis which was carried out within 6 months after the harvest.

### 2.3. Sample Preparation

A suitable amount of each raw rice sample was homogeneously powdered in a laboratory grinder and stored at 4 °C in the dark until extraction and analysis. The rice powder was used for the determination of total phenol index, total flavanols, total anthocyanins and antioxidant capacity (see Section 2.4, Section 2.5 and Section 2.6).

Only the whole rice grain sample of the Venere cultivar (batch 12) was subjected to different types of cooking following the methods described in previous studies. The details are shown in Table 1. After cooking, the rice was weighted to determine water adsorption, then cooled in an ice bath for 20 min, separated from the eventual remaining water and homogenized by a high-speed Ultra-Turrax T25 (IKA^®^-Werke GmbH & Co. KG, Staufen, Germany) before extraction procedures. Moreover, the rice samples selected for their content of bioactive compounds (6 and 13 being Venere and Artemide, respectively) and the Carnaroli one were cooked following the procedure described by Zaupa et al. [6], with a water bath and with different additions of water in a rice cooker.

### 2.4. Extraction Procedure

Both raw and cooked rice samples were suspended in the proper extraction solvent (see below), vortexed for 2 min, sonicated for 10 min, centrifuged at 5000× *g* for 10 min at 4 °C in a bench top centrifuge (Hettich, Tuttlingen, Germany) and the supernatant was recovered. This procedure was repeated 2 times; the recovered supernatants were jointly collected and the volume was adjusted at 5 mL with the same solvent used for the extraction, and tested.

### 2.5. Determination of Phenolic Compounds

Total phenol index (TPI) was determined for the methanolic extracts (methanol/water, 50/50, *v/v*) following the Folin–Ciocalteau method [19] and expressed as g gallic acid/kg. Total flavonoids and anthocyanins were spectrophotometrically quantified in the extracts obtained with hydrochloric ethanol (ethanol/water/hydrochloric acid 37%, 70/30/1, *v/v/v*) as described by Di Stefano et al. [20] and further modifications [21]. Data were expressed as g catechin/kg and g Cy-3glc/kg for total flavanols and total anthocyanins, respectively. The molar extinction coefficients (ε) were 3523 L/mol cm for catechin and 33,414 L/mol cm for Cy-3glc; both were dissolved in hydrochloric ethanol. Detection limits were 1 mg/L for catechin and cyanidin 3-*O*-glucoside. Results were referred to as dried weight for both raw and cooked rice samples.

TPI, total flavonoids and total anthocyanins were also determined in the water remaining after cooking, and diluted in 50% methanol and hydrochloric ethanol, respectively, for TPI, and total flavonoids and total anthocyanins. Data were expressed as mg/L of cooking water.

### 2.6. Determination of Antioxidant Activity

The antioxidant activity of methanolic extracts (methanol/water 70/30 *v/v*) was determined using means of DPPH and ABTS assays.

DPPH assay was carried out following the method of Brand-Williams et al. [22] with some modifications as reported by Fracassetti et al. [21]. The ABTS radical cation scavenging capacity was determined according to the method described by Vitalini et al. [23]. Data were expressed as mM Trolox/kg of dried weight for both raw and cooked rice samples.

### 2.7. Statistical Analysis

Statistical analysis was carried out using SPSS statistical software (IBM SPSS Statistics 24). Analysis of variance (ANOVA) was performed after evaluating the homogeneity of the variance with the Levene test and significant differences between samples were determined with the Fisher test (least significant difference, LSD). Differences were significant for *p* < 0.05.

## 3. Results and Discussion

### 3.1. Bioactive Compounds and Antioxidant Capacity of Pigmented Rice Cultivars

**Figure 1:**
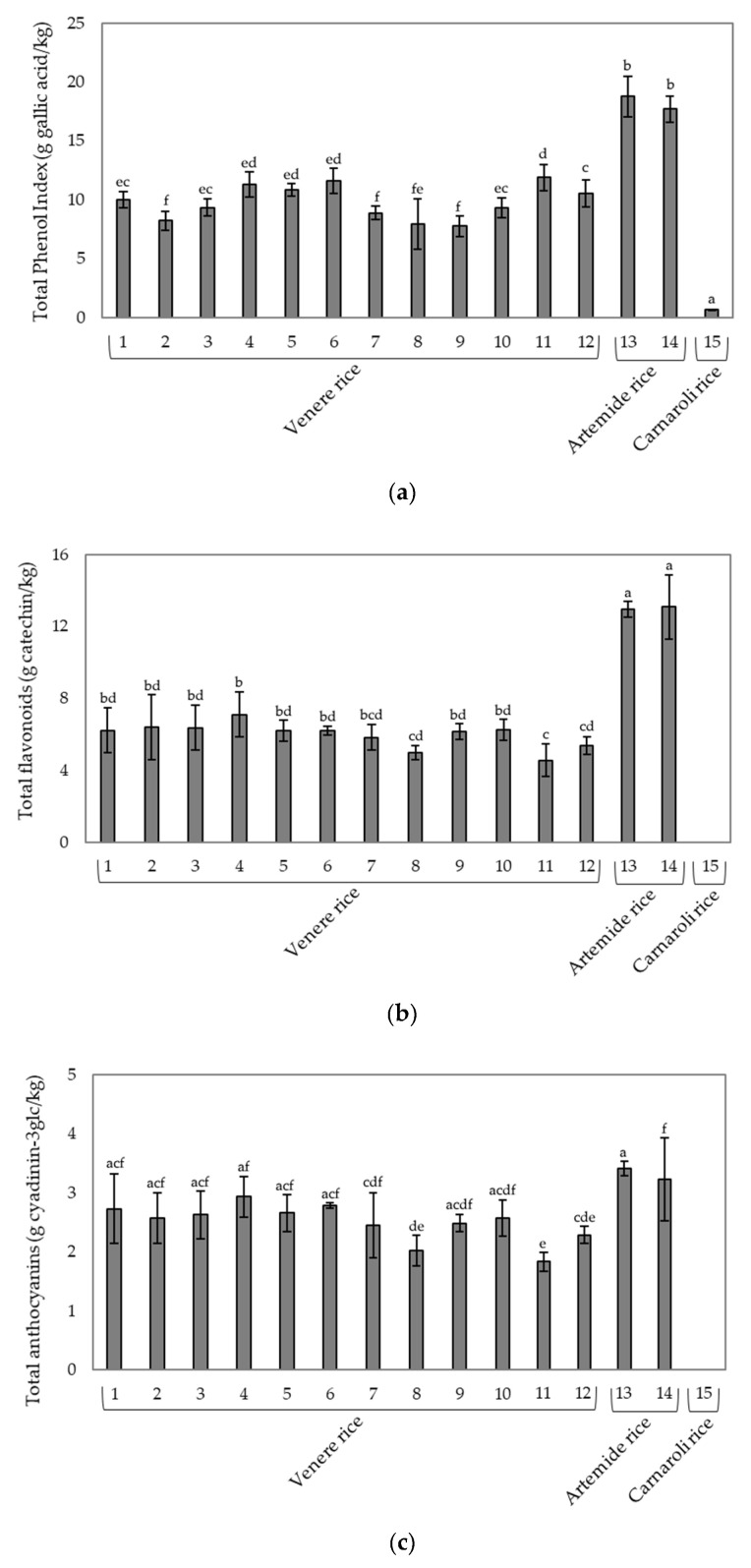
Phenolic compounds in raw rice cultivars. (**a**) Total phenol index (TPI); (**b**) total flavonoids; (**c**) total anthocyanins. Different letters mean significant differences (*p* < 0.05).

Phenolic compounds are synthesized in rice in response to ecological and physiological stresses on the plant, including pathogens, insects and ultraviolet radiation [24,25]. Phenols present in rice include phenolic acids, flavonoids, condensed tannins (proanthocyanidins), lignins and lignans [26]. In rice, flavonoids can be divided into several subgroups, such as flavones, flavonols, flavanones and isoflavones [27]. Among phenolic compounds, the anthocyanins are molecules found in many pigmented plant foods, such as fruit, vegetables and cereals, contributing to their color. In black rice, anthocyanins are located in the aleuronic layer, mainly in the form of cyanidin 3-*O*-glucoside and peonidine 3-glucoside [28,29]. Figure 1 shows the TPI, the contents of total flavonoids and total anthocyanins of the raw rice samples from Venere, Artemide and Carnaroli cultivars. Cultivars with pigmented pericarp were particularly rich in bioactive compounds, with higher contents in the Artemide cultivar than in the Venere cultivar. In detail, TPI was 17.7–18.76 g gallic acid/kg in Artemide rice and it ranged from 8.22 to 11.88 g gallic acid/kg in the Venere rice batches (Figure 1a). These contents agree with those of a recent study conducted by Bordiga et al. [5] that found TPI values of 11.87 ± 1.16 g gallic acid/kg for the Artemide rice and 6.20 ± 0.82 g gallic acid/kg for the Venere rice. In Carnaroli, TPI assay detected a very low amount of phenolics present in the outer layers of the caryopsis. Considering the content of total flavonoids, Artemide rice showed concentration about two-fold higher than Venere rice (13.1 g catechin/kg rice vs. 5.0–7.1 g catechin/kg rice for Artemide and Venere rice, respectively), whereas no flavonoids were detected in Carnaroli rice (Figure 1b). Both pigmented cultivars that resulted were rich in anthocyanins with total contents of 1.83–2.93 g Cy-3glc/kg and 3.23–3.41 g Cy-3glc/kg for Venere and Artemide rice, respectively (Figure 1c). As expected, anthocyanins were not detected in Carnaroli cultivar. The obtained values were higher than those reported in Bordiga et al. [5], in which the total anthocyanin contents were 1.40 ± 0.33 g/kg for Artemide and 0.78 ± 0.17 g/kg for Venere. The differences can be attributed both to the agronomic variability of rice in different years and the different methods used to assess the total anthocyanin content.

**Figure 2:**
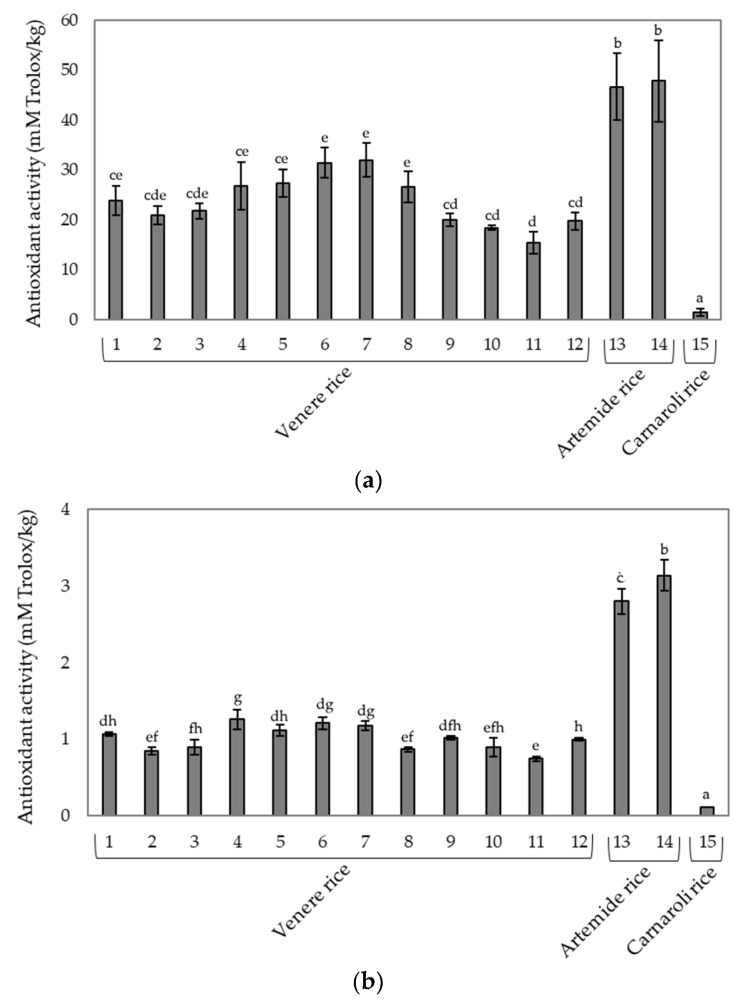
Antioxidant activity in raw rice cultivars determined by (**a**) 2,2-diphenyl-1-picrylhydrazyl (DPPH) assay and (**b**) 2,2′-azino-bis(3-ethylbenzothiazoline-6-sulphonic acid) (ABTS) assay. Different letters mean significant differences (*p* < 0.05).

Phenolic compounds have remarkable antioxidant activity. Interestingly, the latter was higher for anthocyanins than for vitamins C and E [30], with a mechanism able to bind the free radicals related to the donation of hydrogen atoms [31,32]. Since pigmented rice has an interesting phenolic profile, the analysis of the antioxidant activity on the rice samples was carried out. The three tested cultivars showed similar trends against both DPPH and ABTS radical (Figure 2). Nevertheless, Artemide rice was the most effective scavenger with values equal to 47.83 ± 8.1 mM Trolox/kg and 3.14 ± 0.2 mM Trolox/kg, respectively. The obtained values in the DPPH test were greater than those observed by Bordiga et al. [5] (22.8 ± 1.17 mM Trolox/kg). Carnaroli rice showed an antioxidant activity much lower than that found in both Venere and Artemide rice cultivars (Figure 2). This could be expected due to the absence of phenolic compounds, the presence of which is associated to the antioxidant capacity of pigmented rice [4,5,6].

### 3.2. Effect of Cooking on Bioactive Compounds and Antioxidant Capacity

In order to verify the loss of bioactive compounds, Venere 6, Artemide 13 and Carnaroli 15 samples were chosen to be submitted to a water bath cooking test first carried out under laboratory conditions, following the same procedure proposed by Zaupa et al. [6]. After cooking, the TPI decreased to 31.7% for Carnaroli, 58.3% for Venere and 71.7% for the Artemide compared to the contents in the raw samples (Table 2). The losses in our rice cultivars were greater than those reported by Zaupa et al. [6], where the percentage loss of total phenols was 33.5% in water bath cooking and 37.9% in the boiling procedure.

Flavonoid losses were equal to 72.8% and 53.9% in Venere and Artemide cultivars, respectively (Table 2). In the Venere cultivar, the anthocyanin content decreased by 59.1%, while in the Artemide cultivar, the loss was 48.2% (Table 2). In the study of Zaupa et al. [6], both tested heat treatments significantly affected the total anthocyanin content, decreasing it by 30% with water bath cooking and 64% with boiling. A significant reduction of the total anthocyanin content in black rice ranging from 65% to 79% based on the used cooking method was also reported by Hiemori et al. [12].

Table 2 also shows the antioxidant activity of the three selected rice batches. The capacity to scavenge DPPH radical showed percentage increases compared to the raw samples. In detail, 58.5%, 40.9% and 46.5% increases were observed for the Carnaroli, Venere and Artemide cultivars, respectively. This increase was also found by Zaupa et al. [6] which partially related it to the significant increase in protocatecuic acid (+262%) resulting from the degradation of Cy-3glc during heat treatment. On the contrary, the antioxidant activity evaluated with ABTS assay decreased after cooking for both Venere (41.3%) and Artemide (60.4%) cultivars. Such a difference between the results obtained using the two assays could be ascribed to a major decrease, during cooking, of certain phenolic compounds, namely procyanidins, that are preferentially bound with ABTS [33,34].

Afterwards, cooking tests were carried out under real operating conditions. The phenolic compound loss was assessed using total anthocyanins and total flavonoids as markers. The decrease in phenolic compounds can be attributed to several factors. Among them, the main ones can be (i) the water volume-to-rice weight ratio due to the release of bioactive molecules in the cooking water, (ii) the temperature able to influence the stability of compounds such as anthocyanins and (iii) the cooking time.

In order to evaluate the influence of the above mentioned cooking conditions, tests were carried out considering different cooking times, heat source and water volume-to-rice weight ratio (Table 1).

**Figure 3:**
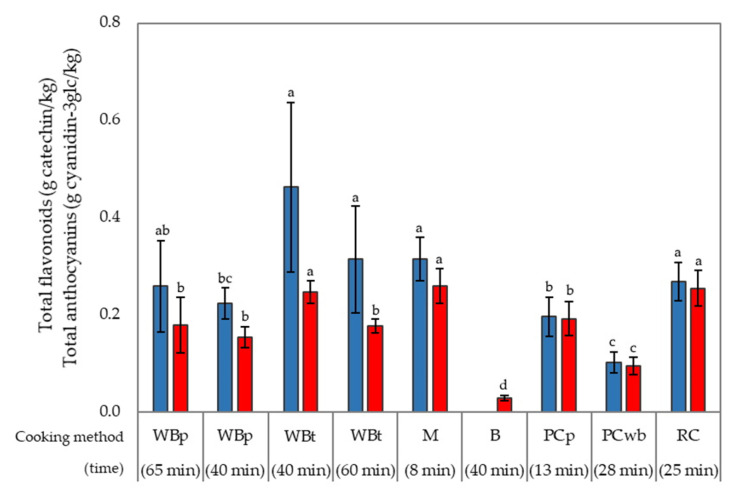
Effect of different cooking methods on total flavonoids (blue) and total anthocyanins (red) for Venere rice (sample 12). Legend: WBp, water bath on plate; WBt, water bath on thermostatic bath; M, microwave; B, boiling; PCp, pressure cooker pilaf method; PCwb, pressure cooker with water bath method; RC, rice cooker. Different letters mean significant differences (*p* < 0.05). The contents of total flavonoids and total anthocyanins in raw rice sample were 5.36 ± 0.49 g catechin/g rice and 2.28 ± 0.15 g cyanidin 3-*O*-glucoside/g; these were significantly different.

Figure 3 summarizes the data obtained for a cooked rice sample of Venere rice (batch 12) prepared using different cooking procedures. The cooking water was also considered when it was left after cooking. Higher contents of both flavonoids and anthocyanins was preserved when pigmented rice was cooked by water bath on thermostatic bath (WBt) for 40 min, microwave (M, 8 min cooking) and rice cooker (RC, 25 min cooking). Rice contained 0.46 ± 0.17, 0.32 ± 0.04 and 0.27 ± 0.04 g catechin/kg and 0.25 ± 0.02, 0.26 ± 0.04, 0.26 ± 0.04 Cy-3glc/kg after cooking in Wbt, M and RC, respectively. Longer cooking time (60 min) under WBt conditions led to a lower content of both flavonoids (0.31 ± 0.11 g catechin/kg) and anthocyanins (0.18 ± 0.02 Cy-3glc/kg), indicating the importance of keeping the cooking time under control to majorly preserve the bioactive compounds. The almost total loss of both flavonoids and anthocyanins was found with the boiling method (B) (−100% for total flavonoids and −98.7% for total anthocyanins), indicating the significant influence of cooking water on the decrease of bioactive compounds [6,11]. When rice was cooked by RC, microwave (M) and pressure cooker (pilaf method, PCp), residual water was still present at the end of the process and it was about 400, 15 and 60 mL, respectively for RC, M and PCp cooking methods. It was separated from the rice and the total flavonoid and anthocyanin content was determined. The results showed high values in water remaining after cooking as 2.21 ± 0.08 g catechin/L and 7.55 ± 0.40 g Cy-3glc/L after RC cooking, 0.17 ± 0.05 g catechin/L and 0.10 ± 0.01 g Cy-3glc/L after M cooking and 2.18 ± 0.01 g catechin/L 1.12 ± 0.01 g Cy-3glc/L in water remaining after PCp cooking. The data show that the lower presence of phenolic compounds in cooked rice could be due to their migration to water during the cooking process, as previously suggested by Towo et al. [17].

**Figure 4:**
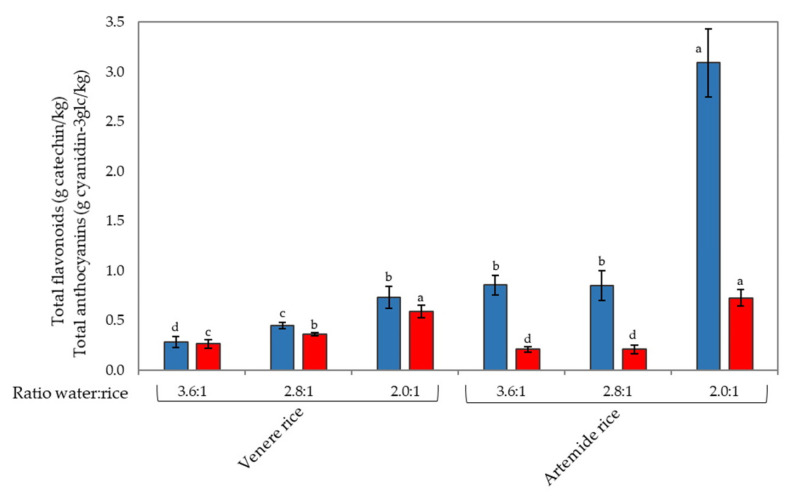
Effect of different water volume-to-rice weight ratio on total flavonoids (blue) and total anthocyanins (red) in Venere (sample 6) and Artemide (sample 13) rice cultivars after cooking in water bath. Different letters mean significant differences (*p* < 0.05).

In order to investigate the role of water, a water bath cooking test was conducted on the selected Venere and Artemide cultivars (samples 6 and 13, respectively). Three different water volume-to-rice weight ratios were used (3.6:1, 2.8:1, 2:1), and total anthocyanin and total flavonoid contents were determined after cooking (Figure 4). The results showed that the 2:1 water volume-to-rice weight ratio better preserved the bioactive compounds in both the Artemide (3.09 ± 0.34 g catechin/g and 0.73 ± 0.08 Cy-3glc/g) and Venere (0.73 ± 0.11 g catechin/g and 0.59 ± 0.06 Cy-3glc/g) cultivars. Furthermore, the levels of flavonoids were about three-fold higher in Artemide rice than in Venere rice (Figure 4).

The contents of total flavonoids and total anthocyanins in Venere rice sample 6 were 5.84 ± 0.69 g catechin/g rice and 2.45 ± 0.55 g cyanidin 3-*O*-glucoside/g. The contents of total flavonoids and total anthocyanins in Artemide rice sample 13 were 12.96 ± 0.44 g catechin/g rice and 3.41 ± 0.12 g cyanidin 3-*O*-glucoside/g. For both rice samples, the data related to raw samples were significantly different in comparison to the cooked samples. Based on the achieved results, the two cooking methods leading to the lower decrease of bioactive compounds and antioxidant capacity were water bath and rice cooker. The two cooking methods showed similar temperature of preparation (around 95 °C) and they differed for the duration of cooking: 40 min for the water bath method and 25 min for rice cooker (Table 1). The two selected cooking procedures were adopted to assess TPI, total flavonoids and total anthocyanins as well as antiradical potential in the three selected rice cultivars (Venere 6, Artemide 13 and Carnaroli 15 samples) (Table 3). After water bath cooking, the phenolic loss expressed as TPI was 92–95% for both pigmented rice cultivars (0.85 ± 0.07 and 0.78 ± 0.10 g gallic acid/kg for Venere and Artemide rice, respectively), while it decreased up to 86–92% with the rice cooker with remarkable effect on Artemide rice (0.86 ± 0.06 and 2.58 ± 0.29 g gallic acid/kg for Venere and Artemide rice cultivars, respectively). Concerning the flavonoids, the two rice cultivars investigated showed a different behavior as the rice cooker decreased their content in Venere (0.27 ± 0.04 vs. 0.46 ± 0.08 mg catechin/kg in rice cooked with water bath), while they were higher in Artemide rice 1.00 ± 0.27 vs. 1.30 ± 0.13 mg catechin/kg in rice cooked with water bath). A slight difference was found for the anthocyanin content among the two tested cooking methods (Table 3). The antioxidant capacity was higher when rice was cooked in the rice cooker, especially for the Artemide one (5.41 ± 0.75 vs. 0.80 ± 0.12 mM Trolox for rice cooked with water bath). The results agree with those reported by Yamuangmorn and Dell [35], indicating that the rice cooker can preserve higher contents of phenolic compounds and, consequently, a greater antioxidant power compared to cooking in a water bath. According to the above, this behavior could be explained by the presence of water in which hydro-soluble phenolic compounds migrate during cooking. The migration of phenolics can also occur when rice is soaked in water prior to cooking [36]. Interestingly, when the rice was prepared by the cooker method, the water was still present at the end of the procedure and it showed considerable amounts of bioactive compounds for both the two pigmented rice cultivars investigated (Table 3), making the rice cooker the cooking method more suitable than water bath.

Lastly, considering the phenolic content both in cooked rice and cooking water, the amounts of bioactive compounds consumed with a portion (100 g) of pigmented rice were estimated by summing up the contents of the investigated indices obtained from rice and water. We assessed that the consumption of Artemide rice can supply 545 mg catechin/100 g and 614 mg Cy-3glc/100 g while that of Venere rice is 240 mg catechin/100 g and 711 mg Cy-3glc/100 g if cooked with the rice cooker method. This amount can lead to possible health benefits, considering the average consumption. As an example, the dietary intake of anthocyanins has been estimated to be around 11.6 ± 1.1 mg/d for individuals older than 20 in the US [37]. The levels of anthocyanins found in a portion of pigmented rice is widely higher than the increased level of anthocyanins recently proposed by China as 50 mg/d [38].

## 4. Conclusions

The results confirmed the presence of bioactive compounds in the pigmented rice. In particular, the Artemide cultivar was richer in bioactive molecules than the Venere cultivar. The cooking process affected their loss with slight differences when laboratory conditions and real cooking conditions were applied. The water bath and rice cooker methods were the two processes leading to the lowest compound losses. Our study suggests the best cooking conditions are achieved by using rice cooking for 25 min in a water:rice proportion of 2:1. In this condition, the water remaining after cooking was found to be a good source of bioactive compounds. Therefore, its consumption together with rice should be recommended in order to increase the uptake of bioactive molecules.

In view of a forthcoming study aimed at investigating the bioavailability of bioactive compounds after the consumption of pigmented rice in healthy volunteers, the rice cooker will be used for its preparation. The residual cooking water will also be administered since it contains a large amount of phenolics migrated during the cooking.

## Figures and Tables

**Table 1:** Cooking methods.

Cooking Method	Procedure	Time (min)	Water:Rice Ratio (v/w)	Reference
Boiling	100 g rice sample was placed in a pot with boiling water	40	20:1	[11]
Microwave	100 g rice sample was placed in a microwave container, added with water	8	2:1	[18]
Pressure cooker	Pilaf method: 100 g rice sample was placed in a pressure pot and covered with water	13	2:1	[12]
	Water bath (bain–marie method): 100 g rice sample and water were added to a glass container, placed in a pressure pot with water bath	28	2:1	
Water bath	100 g rice sample was added with water in a glass container, capped and placed in a water bath (100 °C). The sample was cooked until it reached complete absorption of water, stirring every 10 min	40–65	(i) 2:1 (ii) 2.8:1 (iii) 3.6:1	[6]
	120 mg rice sample was added with water in a glass container, capped and placed in a water bath (100 °C). Cooking method labeled as “laboratory condition”.	40	2:1	
Rice cooker	500 g rice sample was placed in a rice cooker and added with water	25	2:1	[15]

**Table 2:** Total phenol index, total flavonoids, total anthocyanins and antioxidant capacity of Carnaroli (sample 15), Venere (sample 6) and Artemide (sample 13) rice cultivars, cooked in laboratory conditions. Results are means ± standard deviation of three independent sample preparations. The percentage differences between raw and cooked rice are reported in brackets. Different letters mean significant differences (*p* < 0.05).

Sample.		Total Phenol Index (g gallic acid/kg)	Total Flavonoids (g catechin/kg)	Total Anthocyanins (g Cy-3glc/kg)	Antioxidant Activity	
					DPPH (mM Trolox/kg)	ABTS (mM Trolox/kg)
Carnaroli	Raw	0.65 ± 0.05 ^e^	n.d.	n.d.	1.43 ± 0.76 ^e^	0.12 ± 0.00 ^f^
	Cooked	0.44 ± 0.03 ^a^ (−31.7 ± 0.5)	n.d.	n.d.	3.4 ± 2.2 ^a^ (58.5 ± 8.4)	0.12 ± 0.01 ^a^ (no difference)
Venere	Raw	11.61 ± 1.06 ^d^	6.20 ± 0.26 ^d^	2.78 ± 0.04 ^d^	31.42 ± 3.08 ^d^	1.21 ± 0.08 ^e^
	Cooked	4.83 ± 0.7 ^b^ (−58.3 ± 3.0)	1.69 ± 0.29 ^a^ (−72.8 ± 3.5)	1.14 ± 0.16 ^a^ (−59.1 ± 5.2)	44.3 ± 2.5 ^b^ (40.9 ± 5.9)	0.71 ± 0.02 ^b^ (−41.3 ± 2.2)
Artemide	Raw	18.76 ± 1.71 ^c^	12.96 ± 0.44 ^c^	3.41 ± 0.12 ^c^	46.64 ± 6.72 ^b^	2.80 ± 0.16 ^d^
	Cooked	5.31 ± 0.27 ^b^ (−71.7 ± 1.2)	5.97 ± 0.75 ^b^ (−53.9 ± 4.2)	1.76 ± 0.24 ^b^ (−48.2 ± 5.2)	68.3 ± 4.0 ^c^ (46.5 ± 12.8)	1.11 ± 0.06 ^c^ (−60.4 ± 0.1)

Data are expressed as dried weight. Legend: n.d., not detected; Cy-3glc, cyanidin 3-*O*-glucoside.

**Table 3:** Total anthocyanins and flavonoids content, total polyphenol index (TPI) and antioxidant capacity of Venere, Artemide and Carnaroli rice, cooked by rice cooker and water bath.

Sample	Cooking Method		Total Phenol Index (g gallic acid)	Total Flavonoids (g catechin)	Total Anthocyanins (g Cy-3glc)	Antioxidant Activity	
						DPPH (mM Trolox)	ABTS (mM Trolox)
Carnaroli	Raw samples		0.65 ± 0.05 ^g^	n.d.	n.d.	1.43 ± 0.76 ^e^	0.12 ± 0.00 ^f^
	Water bath	Rice	0.08 ± 0.00 ^a^	n.d.	n.d.	n.d.	0.09 ± 0.00 ^a^
	Rice cooker	Rice	0.14 ± 0.01 ^b^	n.d	n.d.	n.d.	0.01 ± 0.00 ^a^
		Water	n.d.	n.d	n.d.	n.d.	n.d.
Venere	Raw samples		11.61 ± 1.06 ^e^	6.20 ± 0.26 ^d^	2.78 ± 0.04 ^b^	31.42 ± 3.08 ^c^	1.21 ± 0.08 ^b^
	Water bath	Rice	0.85 ± 0.07 ^c^	0.46 ± 0.08 ^a^	0.35 ± 0.07 ^a^	0.66 ± 0.09 ^a^	0.78 ± 0.03 ^b^
	Rice cooker	Rice	0.86 ± 0.06 ^c^	0.27 ± 0.04 ^b^	0.26 ± 0.04 ^a^	0.77 ± 0.30 ^a^	0.29 ± 0.00 ^d^
		Water	3.34 ± 0.24 ^A^	2.17 ± 0.08 ^A^	7.55 ± 0.40 ^A^	6.07 ± 0.52 ^A^	23.62 ± 0.74 ^A^
Artemide	Raw samples		18.76 ± 1.71 ^f^	12.96 ± 0.44 ^e^	3.41 ± 0.12 ^b^	46.64 ± 6.72 ^d^	2.80 ± 0.16 ^d^
	Water bath	Rice	0.78 ± 0.10 ^c^	1.00 ± 0.27 ^b^	0.31 ± 0.07 ^a^	0.80 ± 0.12 ^a^	1.05 ± 0.02 ^b^
	Rice cooker	Rice	2.58 ± 0.29 ^d^	1.30 ± 0.13 ^c^	0.35 ± 0.07 ^a^	5.41 ± 0.75 ^b^	0.41 ± 0.02 ^c^
		Water	4.62 ± 0.52 ^B^	4.58 ± 0.11 ^B^	8.11 ± 0.20 ^A^	22.07 ± 4.20 ^B^	66.38 ± 5.34 ^B^

Data are expressed as dried weight for rice (kg) and in litres for water. Different lowercase letters mean significant differences related to dried weight for rice (*p* < 0.05). Different capital letters mean significant differences related to cooking water (*p* < 0.05). Legend: n.d., not detected; Cy-3glc, cyanidin 3-*O*-glucoside.
